# Supplementary material for: Exposure to images showing (non)adherence to physical distancing rules: Effect on adherence behavior and perceived social norms
Source: PLoS One. 2022 Nov 2;17(11):e0276936. doi: 10.1371/journal.pone.0276936 (PMC9629595; doi:10.1371/journal.pone.0276936)
Supplement: S1 File — S1 Results: S1 Fig. Participant flowchart. S1 Table. Detailed descriptions of the participant sample (n = 315). S2 Table. Robustness analyses (n = 315). We repeated the analyses for number of shoppers using ordered logit regressions. S3 Table. Component paths of the indirect effect of condition on adherence behavior for perceptions of descriptive norms and perceptions of injunctive norms (n = 315). S4 Table. Pairwise correlations (n = 315). (DOCX) [file pone.0276936.s001.docx]

**S1 File**

**S1 Methods**

**Translated instructions online behavioral task**

In this part of the research you will complete an assignment with which you can earn additional money. In this assignment, the aim is to move a stick figure from the left to the right side of the screen. **It is important that you read the information below carefully.**

The assignment starts when you click on the game screen. At that moment, your stick figure enters a supermarket where other people are also shopping. Your stick figure stops at 1.5 meters from other customers when they stand still in your walking route. After some time, those customers will automatically move backwards to make room.

The stick figure will wait by itself. Once your stick figure has stopped, you can click on the orange **‘WALK’** button at the bottom of the screen to continue walking. You can choose to wait before walking until the other customers have made room or to continue walking earlier.

The rule is to wait before walking until it is possible to keep 1.5 meters distance from other customers. It is up to you to decide how you deal with this rule.

How much money you earn in this assignment depends on the time you need to walk our stick figure from the left to the right side of the supermarket. You begin with a starting amount of **€4.-.** For every second it takes you to complete the assignment, **€0.04 will be deducted from the starting amount.**

**Below you will see a number of pictures so that you can see how the supermarket assignment works.**

When the assignment is loaded, you will see the screen below. The assignment starts when you click the gray area of the screen. The stick figure with the shopping cart then automatically starts walking.

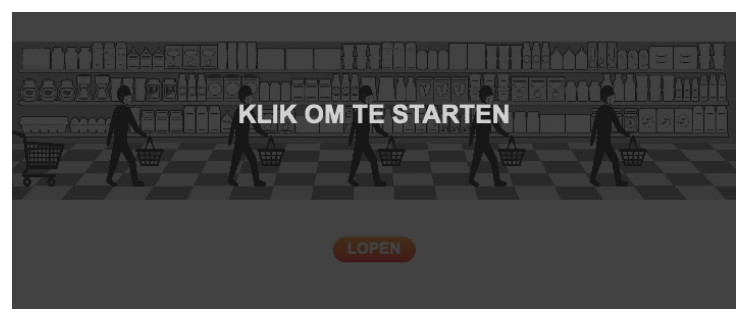

[Translation of text in the picture: Click to start]

The other customers stand still in the walking route of your stick figure. As soon as the stick figure encounters another customer, it automatically stops walking.

Your stick figure, indicated below by way of illustration with the orange circle, has a shopping cart and a black arrow above its head.

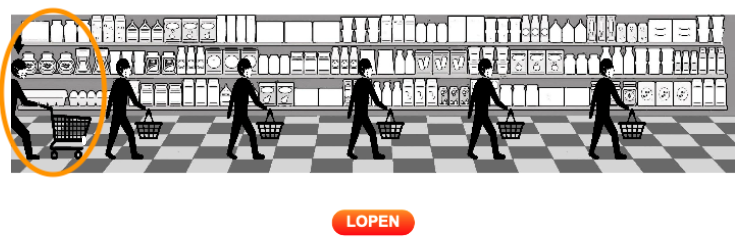

[Translation of text in the picture: WALK]

The customer closest to the stick figure moves backwards to make room.

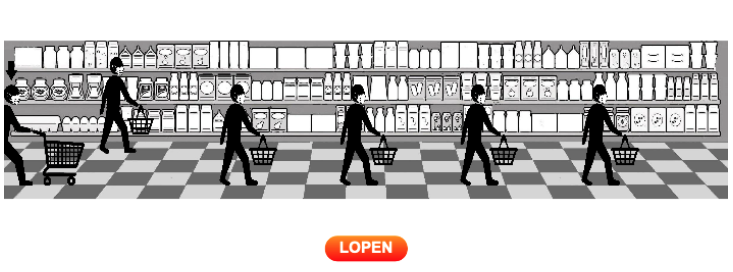

[Translation of text in the picture: WALK]

The stick figure will start walking when the orange “WALK” button is clicked.

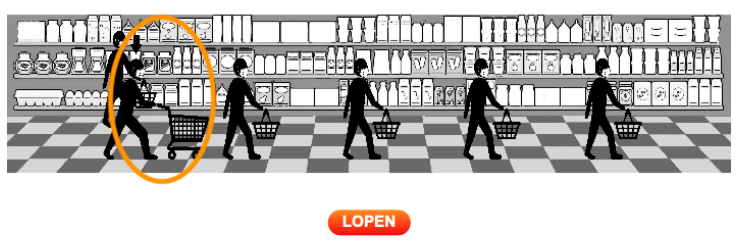

[Translation of text in the picture: WALK]

When the stick figure exits on the right side of the screen, the assignment is over. You can then click on the arrow button to continue.


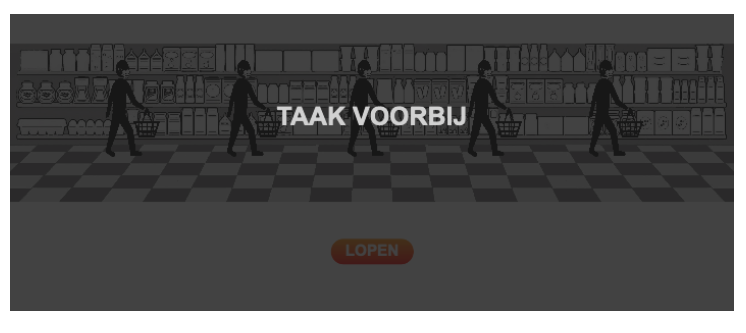


[Translation of text in the picture: Task over]

Have you read the instructions and are ready to do the task yourself, then click on the arrow button.

**S1 Results**


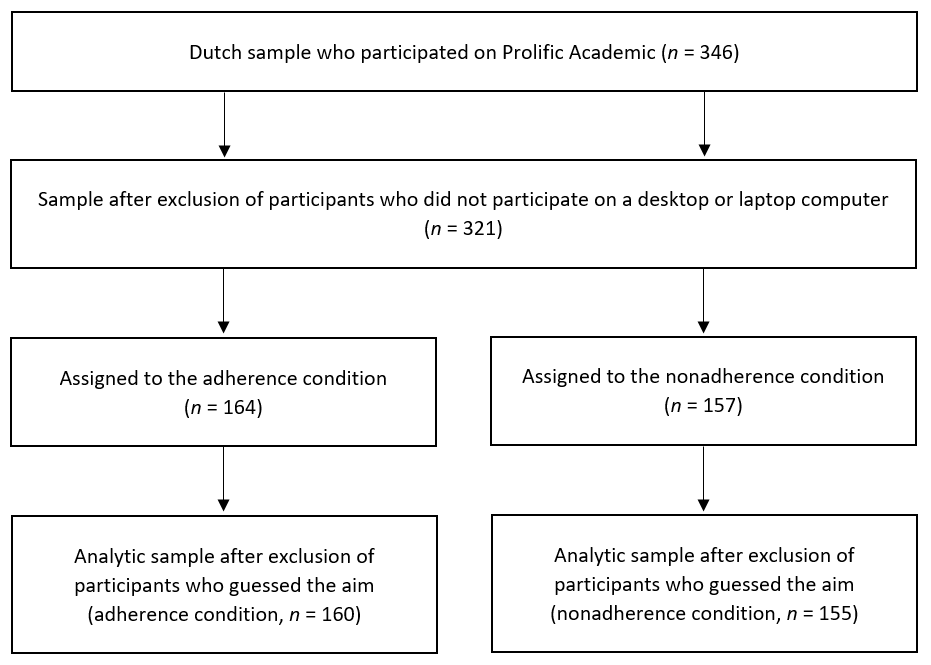
 **S1 Fig. Participant flowchart.**

**S1 Table. Detailed descriptions of the participant sample (*n* = 315).**

|  | **Mean (SD) or Number (%)** |
| --- | --- |
| Age ^b^ (y) | 28.27 (9.53) |
| Sex |  |
| Male | 183 (58.10%) |
| Female | 130 (41.27%) |
| Other | 2 (0.63%) |
| Nationality |  |
| Dutch | 266 (84.44%) |
| Belgian | 2 (0.63%) |
| German | 1 (0.32%) |
| English (Great Britain and Northern Ireland) | 1 (0.32%) |
| Indonesian | 3 (0.10%) |
| Moroccan | 5 (1.6%) |
| Suriname | 2 (0.63%) |
| Turkish | 4 (1.27%) |
| Other | 31 (9.84%) |
| Education |  |
| Primary | 4 (1.27%) |
| Pre-vocational secondary | 9 (2.86%) |
| Senior general or pre-university secondary | 87 (27.62%) |
| Vocational | 19 (6.03%) |
| Higher vocational | 72 (22.86%) |
| Academic | 124 (39.37%) |
| Incidence rate in municipality | 0.00 (1.00) |
| Outcome expectancies ^a^ | 5.78 (1.35) |
| Risk perception ^a^ | 4.47 (1.77) |
| Changes in occupation ^c^ |  |
| No | 112 (35.56%) |
| Yes, due to restrictions | 63 (20%) |
| Yes, due to infection | 8 (2.54%) |
| Yes, increased work pressure | 36 (11.43%) |
| Yes, work from home | 134 (42.54%) |
| Infection with SARS-CoV-2: Personal |  |
| No | 268 (85.08%) |
| Yes, within last 6 months | 21 (6.67%) |
| Yes, longer than 6 months ago | 22 (6.98%) |
| Do not want to answer | 4 (1.27%) |
| Infection with SARS-CoV-2: Surroundings ^c^ |  |
| No | 28 (8.95%) |
| Yes, immediate family | 66 (20.95%) |
| Yes, extended family | 139 (44.13%) |
| Yes, friends | 170 (53.97%) |
| Yes, acquaintances | 163 (51.75%) |
| Do not want to answer | 2 (0.63%) |
| COVID-19 vaccination status |  |
| Yes, completely vaccinated | 50 (15.87%) |
| Yes, partly vaccinated | 112 (35.56%) |
| No, not yet, I will be vaccinated later | 125 (39.68%) |
| No, not planning to | 22 (6.98%) |
| Do not want to answer | 6 (1.90%) |
| Usage of Dutch digital contact tracing app |  |
| Yes, always turned on | 78 (24.76%) |
| Yes, when I think about it | 17 (5.40%) |
| No | 218 (69.21%) |
| Do not want to answer | 2 (0.63%) |
| Received notification of Dutch digital contact tracing app ^d^ |  |
| Yes | 20 (21.05%) |
| No | 75 (78.95%) |

^a^ Ranging from 1–7. ^b^ *n* = 314 because of missing values. ^c^ multiple answers possible, % of 315 subjects. ^d^ *n* = 95 because question only applies to subjects who use the app.

**S2 Table. Robustness analyses (n = 315). We repeated the analyses for number of shoppers using ordered logit regressions.**

| **Dependent variable** | **Waiting time** | | | **Number of shoppers** | | | **Vignettes** | | | **Intention** | | |
| --- | --- | --- | --- | --- | --- | --- | --- | --- | --- | --- | --- | --- |
| Model | (1) | (2) | (3) | (4) | (5) | (6) | (7) | (8) | (9) | (10) | (11) | (12) |
|  |  |  |  |  |  |  |  |  |  |  |  |  |
| Main treatment effect | 1.056 | 1.228 | 1.213 | 0.168 | 0.218 | 0.200 | 0.094 | 0.110 | 0.077 | 0.087 | 0.127 | 0.065 |
|  | (1.416) | (1.294) | (1.303) | (0.214) | (0.199) | (0.207) | (0.180) | (0.182) | (0.175) | (0.141) | (0.136) | (0.113) |
| Descriptive social norms |  | 2.304*** | 2.193*** |  | 0.437*** | 0.428*** |  | 0.099 | 0.062 |  | 0.174** | 0.118* |
|  |  | (0.489) | (0.507) |  | (0.080) | (0.080) |  | (0.082) | (0.078) |  | (0.062) | (0.047) |
| Injunctive social norms: Item 1 |  | -0.818 | -1.007* |  | -0.177* | -0.223** |  | -0.031 | -0.079 |  | 0.012 | -0.040 |
|  |  | (0.506) | (0.499) |  | (0.081) | (0.081) |  | (0.071) | (0.065) |  | (0.055) | (0.042) |
| Injunctive social norms: Item 2 |  | 0.491 | 0.317 |  | 0.196* | 0.178* |  | 0.058 | 0.076 |  | 0.086 | 0.091+ |
|  |  | (0.659) | (0.764) |  | (0.080) | (0.079) |  | (0.082) | (0.079) |  | (0.055) | (0.049) |
| Age |  |  | 0.030 |  |  | 0.004 |  |  | 0.031** |  |  | 0.013* |
|  |  |  | (0.059) |  |  | (0.010) |  |  | (0.011) |  |  | (0.006) |
| Sex (male) |  |  | -2.283+ |  |  | -0.344+ |  |  | -0.379* |  |  | -0.133 |
|  |  |  | (1.304) |  |  | (0.205) |  |  | (0.177) |  |  | (0.112) |
| Nationality (Dutch) |  |  | -0.696 |  |  | -0.065 |  |  | 0.023 |  |  | -0.217 |
|  |  |  | (2.036) |  |  | (0.290) |  |  | (0.218) |  |  | (0.138) |
| Education (academic education) |  |  | -1.527 |  |  | -0.074 |  |  | 0.021 |  |  | -0.128 |
|  |  |  | (1.372) |  |  | (0.216) |  |  | (0.179) |  |  | (0.113) |
| Incidence rate in municipality |  |  | -0.443 |  |  | -0.089 |  |  | -0.079 |  |  | -0.060 |
|  |  |  | (0.615) |  |  | (0.099) |  |  | (0.088) |  |  | (0.059) |
| Outcome expectancies |  |  | 1.313* |  |  | 0.190* |  |  | 0.436*** |  |  | 0.575*** |
|  |  |  | (0.591) |  |  | (0.095) |  |  | (0.066) |  |  | (0.060) |
| Risk perception |  |  | 0.213 |  |  | -0.037 |  |  | 0.003 |  |  | -0.056+ |
|  |  |  | (0.460) |  |  | (0.067) |  |  | (0.048) |  |  | (0.031) |
| Changes in occupation due to COVID-19 (no) |  |  | -0.810 |  |  | 0.195 |  |  | -0.142 |  |  | -0.202+ |
|  |  |  | (1.461) |  |  | (0.223) |  |  | (0.181) |  |  | (0.111) |
| Infection with COVID-19: Personal (yes) |  |  | 2.595+ |  |  | 0.437+ |  |  | -0.131 |  |  | -0.150 |
|  |  |  | (1.493) |  |  | (0.254) |  |  | (0.259) |  |  | (0.169) |
| Infection with COVID-19: Surroundings (yes) |  |  | 1.110 |  |  | 0.066 |  |  | -0.571 |  |  | -0.272 |
|  |  |  | (2.362) |  |  | (0.372) |  |  | (0.365) |  |  | (0.201) |
| COVID-19 vaccination status (yes) |  |  | -1.395 |  |  | -0.025 |  |  | -0.158 |  |  | -0.003 |
|  |  |  | (1.555) |  |  | (0.223) |  |  | (0.184) |  |  | (0.121) |
| Usage of Dutch digital contact tracing app (yes) |  |  | 1.527 |  |  | 0.356 |  |  | 0.096 |  |  | 0.081 |
|  |  |  | (1.645) |  |  | (0.223) |  |  | (0.194) |  |  | (0.121) |
| Constant | 23.324*** | 15.482*** | 10.011* | 3.619*** | 1.696*** | 1.032 | 4.119*** | 3.568*** | 1.290+ | 5.706*** | 4.491*** | 2.174*** |
|  | (0.897) | (4.050) | (4.504) | (0.155) | (0.467) | (0.790) | (0.124) | (0.403) | (0.722) | (0.101) | (0.312) | (0.459) |
| Observations | 315 | 315 | 305 | 315 | 315 | 305 | 315 | 315 | 305 | 315 | 315 | 305 |
| R-squared | 0.002 | 0.081 | 0.116 | 0.002 | 0.157 | 0.200 | 0.001 | 0.015 | 0.175 | 0.001 | 0.093 | 0.454 |
| F test | 0.556 | 9.719 | 3.744 | 0.616 | 13.66 | 4.597 | 0.274 | 1.037 | 4.257 | 0.382 | 6.633 | 11.79 |
| Prob > F | 0.457 | 2.04e-07 | 2.71e-06 | 0.433 | 2.85e-10 | 3.25e-08 | 0.601 | 0.388 | 1.90e-07 | 0.537 | 3.92e-05 | 0 |

Robust standard errors in parentheses. *** *p* < 0.001, ** *p* < 0.01, * *p* < 0.05, + *p* < 0.1.

**S3 Table. Component paths of the indirect effect of condition on adherence behavior for perceptions of descriptive norms and perceptions of injunctive norms** **(*n* = 315).**

|  |  | **Condition – proposed mediator relationship** | **Proposed mediator – adherence behavior relationship** |
| --- | --- | --- | --- |
|  |  | ***B* (95% CI)** | ***B* (95% CI)** |
| Perceptions of descriptive norms | Online behavioral task | -0.11 (-0.47, 0.26) |  |
|  | Waiting time (seconds) |  | 2.04*** (1.29, 2.82) |
|  | Number of shoppers waited for |  | 0.42*** (0.29, 0.55) |
|  | Intention to adhere |  |  |
|  | Vignettes |  | 0.11+ (-0.01, 0.22) |
|  | Single item measure |  | 0.22*** (0.13, 0.31) |
| Perceptions of injunctive norms:  Item 1 | Online behavioral task | -0.22 (-0.59, 0.15) |  |
|  | Waiting time (seconds) |  | 0.71 (-0.40, 1.80) |
|  | Number of shoppers waited for |  | 0.16* (0.02, 0.30) |
|  | Intention to adhere |  |  |
|  | Vignettes |  | 0.05 (-0.06, 0.16) |
|  | Single item measure |  | 0.15** (0.06, 0.24) |
| Perceptions of injunctive norms:  Item 2 | Online behavioral task | -0.21 (-0.55, 0.13) |  |
|  | Waiting time (seconds) |  | 1.22+ (-0.16, 2.60) |
|  | Number of shoppers waited for |  | 0.32*** (0.17, 0.47) |
|  | Intention to adhere |  |  |
|  | Vignettes |  | 0.09 (-0.04, 0.22) |
|  | Single item measure |  | 0.18*** (0.09, 0.27) |

*B*  = unstandardized regression coefficient. CI = robust confidence interval. *** *p* < 0.001, ** *p* < 0.01, * *p* < 0.05, + *p* < 0.1.

**S4 Table. Pairwise correlations (n = 315).**

| Variable | 1. | 2. | 3. | 4. | 5. | 6. | 7. | 8. | 9. | 10. | 11. | 12. | 13. | 14. | 15. | 16. | 17. | 18. | 19. |
| --- | --- | --- | --- | --- | --- | --- | --- | --- | --- | --- | --- | --- | --- | --- | --- | --- | --- | --- | --- |
| 1. Waiting time (seconds) | - |  |  |  |  |  |  |  |  |  |  |  |  |  |  |  |  |  |  |
| 2. Number of shoppers waited for | 0.745^***^ | - |  |  |  |  |  |  |  |  |  |  |  |  |  |  |  |  |  |
| 3. Vignettes | 0.141^*^ | 0.218^***^ | - |  |  |  |  |  |  |  |  |  |  |  |  |  |  |  |  |
| 4. Single item measure | 0.259^***^ | 0.309^***^ | 0.526^***^ | - |  |  |  |  |  |  |  |  |  |  |  |  |  |  |  |
| 5. Descriptive norms | 0.258^***^ | 0.360^***^ | 0.115^*^ | 0.279^***^ | - |  |  |  |  |  |  |  |  |  |  |  |  |  |  |
| 6. Injunctive norms: Item 1 | 0.0805 | 0.129^*^ | 0.0563 | 0.184^**^ | 0.562^***^ | - |  |  |  |  |  |  |  |  |  |  |  |  |  |
| 7. Injunctive norms: Item 2 | 0.129^*^ | 0.242^***^ | 0.0807 | 0.199^***^ | 0.472^***^ | 0.499^***^ | - |  |  |  |  |  |  |  |  |  |  |  |  |
| 8. Age (y) | -0.0204 | -0.00838 | 0.127^*^ | 0.0277 | 0.0648 | 0.0741 | -0.0601 | - |  |  |  |  |  |  |  |  |  |  |  |
| 9. Sex (male) | -0.0737 | -0.0946^+^ | -0.127^*^ | -0.0778 | -0.0299 | -0.0735 | -0.0644 | -0.0523 | - |  |  |  |  |  |  |  |  |  |  |
| 10. Nationality (Dutch) | -0.0197 | -0.00818 | 0.0241 | -0.0288 | -0.0518 | 0.0440 | 0.0859 | 0.0253 | -0.0289 | - |  |  |  |  |  |  |  |  |  |
| 11. Education (academic) | -0.0591 | -0.0363 | 0.0490 | 0.00919 | -0.0586 | 0.0213 | -0.0362 | 0.112^+^ | -0.0879 | -0.0496 | - |  |  |  |  |  |  |  |  |
| 12. Municipal incidence rate | -0.0307 | -0.0548 | -0.0218 | -0.0298 | -0.0449 | -0.0909 | -0.0433 | -0.00467 | -0.0256 | -0.0972^+^ | 0.0573 | - |  |  |  |  |  |  |  |
| 13. Outcome expectancies | 0.169^**^ | 0.181^**^ | 0.330^***^ | 0.613^***^ | 0.152^**^ | 0.163^**^ | 0.105^+^ | -0.134^*^ | -0.0410 | 0.0422 | 0.0739 | 0.0425 | - |  |  |  |  |  |  |
| 14. Risk perception | 0.00666 | -0.0633 | -0.00873 | -0.0758 | -0.0506 | -0.0368 | -0.0252 | -0.106^+^ | 0.0936 | -0.0140 | 0.0193 | 0.138^*^ | 0.0425 | - |  |  |  |  |  |
| 15. Changes in occupation (no) | 0.00959 | 0.0847 | -0.0424 | -0.0543 | 0.0912 | 0.103^+^ | 0.0879 | -0.0824 | -0.0699 | -0.0258 | -0.199^***^ | -0.00782 | -0.00298 | -0.0212 | - |  |  |  |  |
| 16. Infection with SARS-CoV-2: Personal (yes) | 0.0748 | 0.0857 | -0.0410 | -0.0499 | 0.0399 | 0.124^*^ | 0.0778 | -0.0234 | -0.0376 | 0.0351 | 0.0198 | -0.0422 | -0.00266 | 0.0256 | 0.0499 | - |  |  |  |
| 17. Infection with SARS-CoV-2: Surroundings (yes) | 0.0546 | 0.0614 | -0.0548 | 0.0437 | 0.0200 | 0.0738 | 0.0947^+^ | -0.0803 | -0.00568 | 0.0588 | 0.0363 | -0.0584 | 0.158^**^ | -0.122^*^ | -0.0106 | 0.123^*^ | - |  |  |
| 18. COVID-19 vaccination status (yes) | -0.0382 | -0.00107 | 0.0569 | 0.0804 | -0.00181 | 0.0376 | -0.0438 | 0.351^***^ | -0.0982^+^ | 0.0483 | 0.196^***^ | -0.0274 | 0.0802 | 0.0141 | -0.146^*^ | -0.0483 | 0.0500 | - |  |
| 19. Usage of Dutch digital contact tracing app (yes) | 0.0531 | 0.0837 | 0.0400 | 0.0792 | -0.0104 | -0.00852 | -0.0485 | -0.0546 | 0.0741 | 0.108^+^ | 0.0775 | -0.0415 | 0.110^+^ | -0.0584 | -0.0636 | -0.0132 | 0.0830 | 0.0809 | - |

*** *p* < 0.001, ** *p* < 0.01, * *p* < 0.05, + *p* < 0.1.
